# Supplementary figures and images for: Age influences on Propofol estimated brain concentration and entropy during maintenance and at return of consciousness during total intravenous anesthesia with target-controlled infusion in unparalyzed patients: An observational prospective trial
Source: PLoS One. 2020 Dec 22;15(12):e0244145. doi: 10.1371/journal.pone.0244145 (PMC7755218; doi:10.1371/journal.pone.0244145)

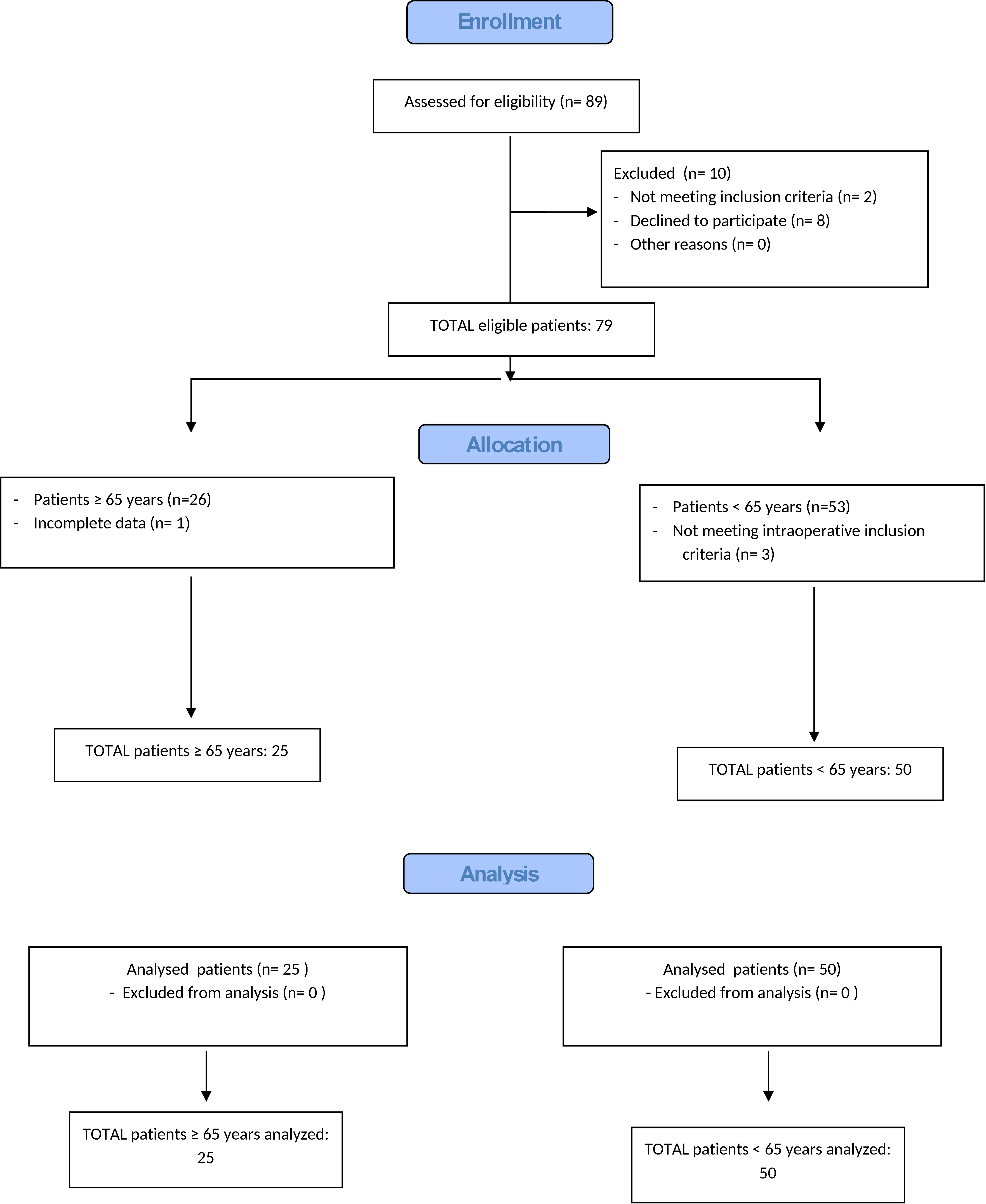

Supplement: S1 Fig — (TIF) [file pone.0244145.s001.tif]

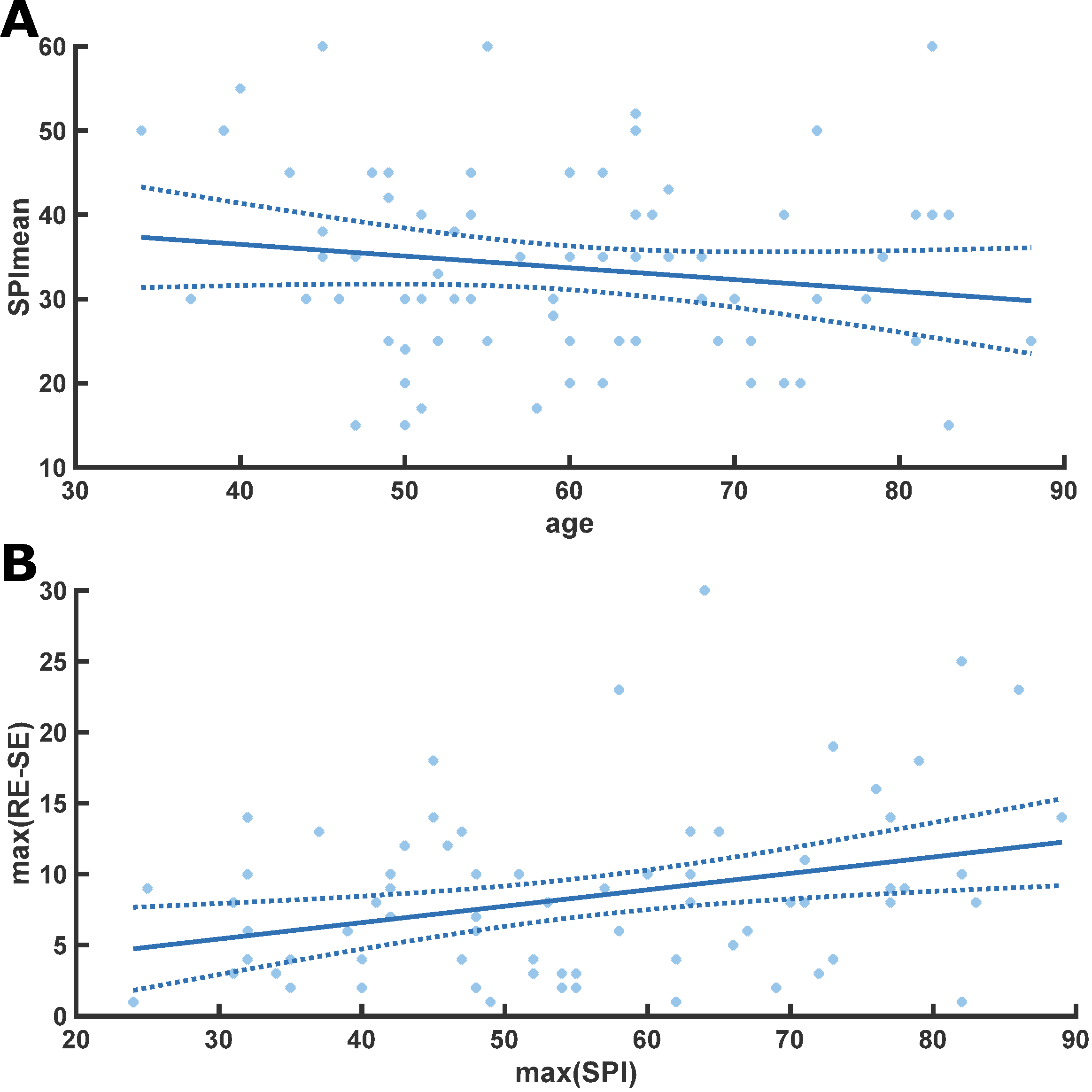

Supplement: S2 Fig — Scatter plots with linear regression for the A) mean SPI versus age (years) and B) max(SPI) versus max(RE-SE) relationship during anesthesia maintenance. There was no significant change of the mean SPI with age. There was a significant increase of the max (RE-SE) with increase of the maximum SPI observed during anesthesia maintenance. SPI: Surgical Pleth Index; RE: Response entropy; SE: State Entropy. (TIF) [file pone.0244145.s002.tif]

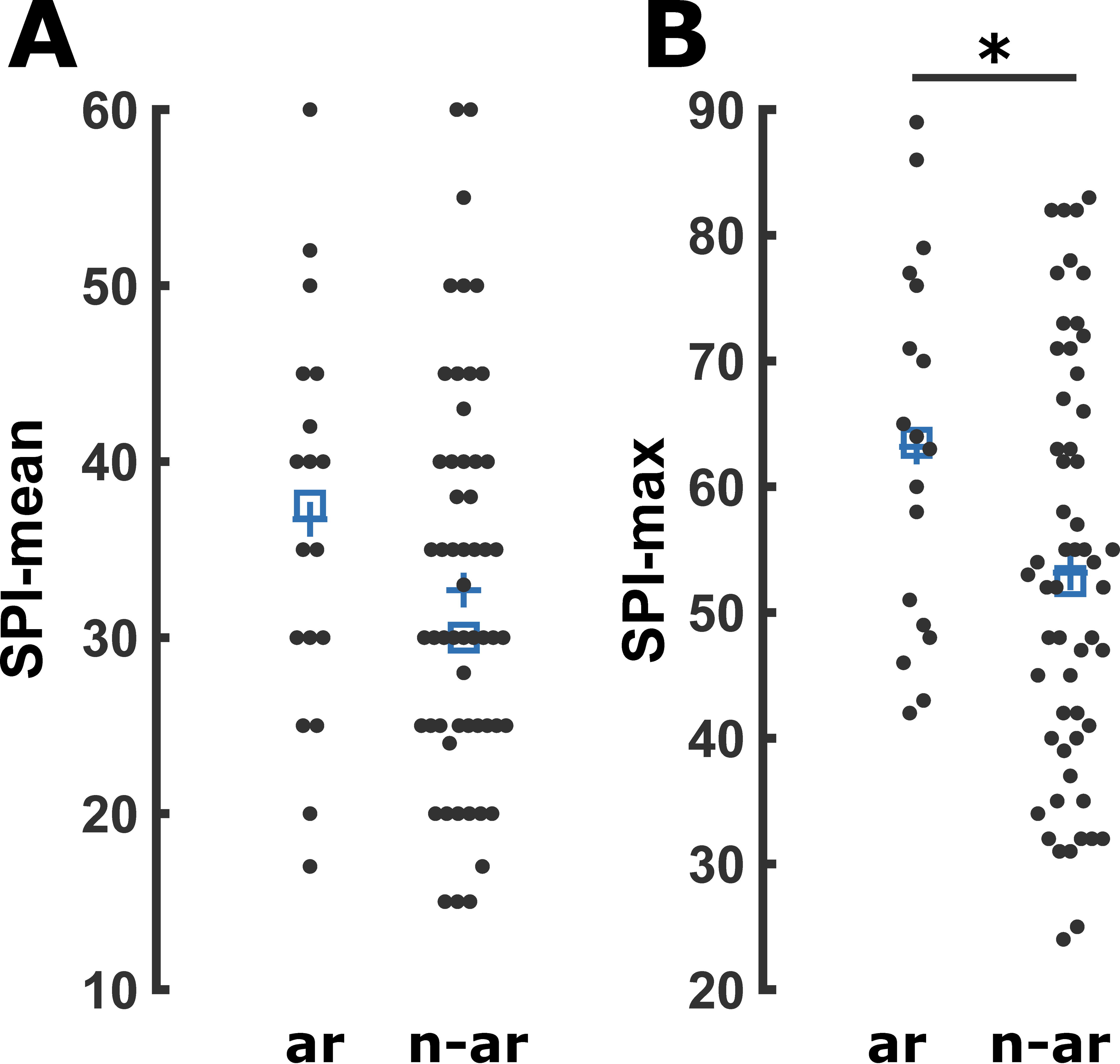

Supplement: S3 Fig — There was no significant difference of mean SPI between arousers and non-arousers during anesthesia maintenance. The maximum SPI was significantly higher in arouser during anesthesia maintenance (p = 0.03; AUC = 0.67 [0.53–0.80]). SPI: Surgical Pleth Index; ar: arousers; n-ar: non-arousers. (TIF) [file pone.0244145.s003.tif]

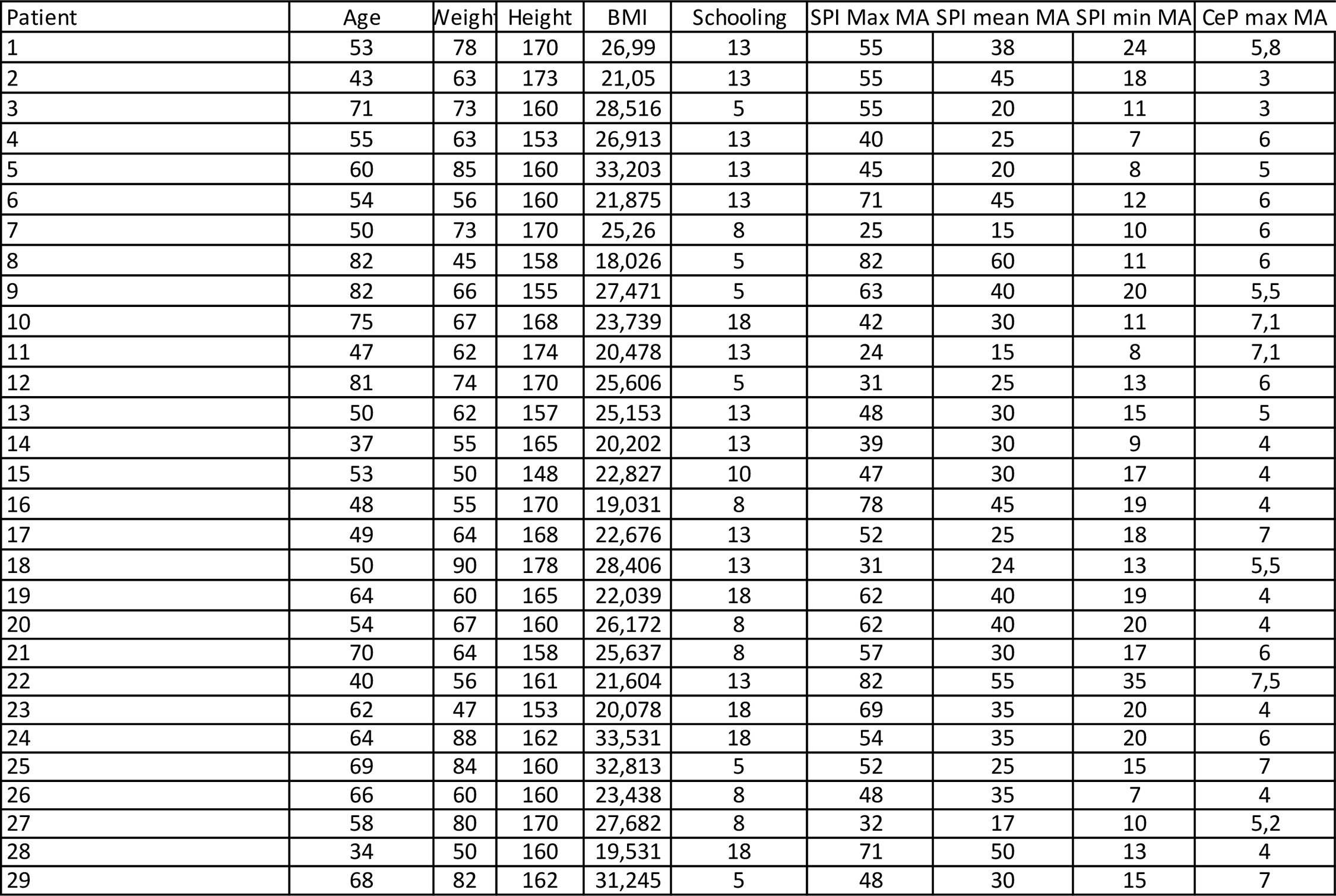

Supplement: S1 Database — (TIF) [file pone.0244145.s004.tif]
